# Genetic Variation of Kallikrein-Kinin System and Related Genes in Patients With Hereditary Angioedema

**DOI:** 10.3389/fmed.2019.00028

**Published:** 2019-02-21

**Authors:** Camila Lopes Veronez, Anne Aabom, Renan Paulo Martin, Rafael Filippelli-Silva, Rozana Fátima Gonçalves, Priscila Nicolicht, Agatha Ribeiro Mendes, Jane Da Silva, Mar Guilarte, Anete Sevciovic Grumach, Eli Mansour, Anette Bygum, João Bosco Pesquero

**Affiliations:** ^1^Department of Biophysics, Federal University of São Paulo, São Paulo, Brazil; ^2^Department of Dermatology and Allergy Centre, Odense University Hospital, Odense, Denmark; ^3^Institute of Genetic Medicine, Johns Hopkins University, Baltimore, MD, United States; ^4^Private Allergy and Immunology Clinic, Belo Horizonte, Brazil; ^5^Department of Medicine, Allergy Clinic of Professor Polydoro Ernani de São Thiago University Hospital, Federal University of Santa Catarina, Florianopolis, Brazil; ^6^Allergy Section, Internal Medicine Department, Hospital Universitari Vall d'Hebron, Barcelona, Spain; ^7^Division of Clinical Immunology, Faculty of Medicine ABC, Santo André, Brazil; ^8^Department of Internal Medicine, University of Campinas, Campinas, Brazil

**Keywords:** hereditary angioedema, Kallikrein-Kinin System, genetic variation, genotype-phenotype correlation, C1 inhibitor deficiency, *F12* mutation

## Abstract

Hereditary angioedema (HAE) is an autosomal dominant disease caused by C1-INH deficiency due to mutations in *SERPING1* (C1-INH-HAE) in most of the cases, or by specific mutations in factor XII gene, *F12* (F12-HAE). Identification of polymorphisms in the genes encoding proteins from key pathways driving HAE can help to understand how genetic diversity contributes to its phenotypic variability. Here, 15 genes related to the Kallikrein-Kinin System (KKS) were analyzed by next generation sequencing in 59 patients with C1-INH-HAE or F12-HAE from Brazil, Denmark and Spain, and 19 healthy relatives in a total of 31 families. We identified 211 variants, from which 23 occurred only in Danish subjects and 79 were found only in Brazilian individuals, resulting in 109/211 variations in common between European and Brazilian population in the HAE families analyzed. *BDKRB2* and CPM presented a large number of variants in untranslated regions, 46/49 and 19/24, respectively; whereas *ACE* (*n* = 26), *SERPING1* (*n* = 26), *CPM* (*n* = 24), and *NOS3* (*n* = 16) genes presented the higher number of variants directly affecting amino acid sequence. Despite the large amount of variants identified, the lack of association between genotype and phenotype indicates that the modulation of HAE symptom requires a more complex regulation, probably involving pathways beyond the KKS, epigenetics and environmental factors. Considering the new HAE types recently described, molecules involved in the regulation of vasculature and in plasminogen activation become promising targets for future genetic studies.

## Introduction

Hereditary angioedema (HAE) is a rare autosomal dominant disease caused by deleterious mutations in *SERPING1*, leading to quantitative or functional C1-inhibitor deficiency (C1-INH-HAE) (1). Less frequently, specific mutations in *F12* gene (F12-HAE) can be detected in patients with HAE with normal C1-inhibitor (2, 3). In addition, two new forms of HAE have been reported, HAE with mutations in angiopoietin-1 gene (*ANGPT1*) (4) and HAE with mutations in plasminogen gene (*PLG*) (5). A non-determined but substantial proportion of patients with HAE and normal C1-inhibitor cannot be explained by mutational findings at the moment and therefore is described as HAE of unknown cause (U-HAE) (1, 6). C1-INH-HAE and F12-HAE are related to the Kallikrein-Kinin System (KKS) activation and augmented production of bradykinin (BK), leading to vasodilation and angioedema episodes (1). In the KKS, factor XII (encoded by *F12* gene) activates plasma kallikrein (KK; *KLKB1* gene), which hydrolyzes high molecular weight kininogen (HMWK; *KNG1* gene) releasing BK (7). BK binds the B2-receptor (*BDKRB2* gene) leading to endothelial nitric oxide synthase activation (*NOS3* gene). Carboxypeptidases N and M (genes *CPN* and *CPM*) metabolize BK into des-Arg9-BK, which preferentially activates B1-receptor (*BDKRB1*). Deleterious variations in genes responsible for the control of BK release, as *SERPING1*, or its metabolization, as *CPN, CPM*, angiotensin-converting enzyme (*ACE*), and neprilysin (*MME*), are able to increase the amount of BK released or its half-life, leading to or intensifying angioedema episodes. Whereas, deleterious variations in *F12, KLKB1*, and *KNG1* could impair BK formation, working as a protective factor in BK-mediated angioedema (7). Although a small phenotype distinction can be noticed in HAE subtypes (2), there is a huge variation in clinical characteristics even among carriers of the same disease-causing mutation (1, 6).

A typical genome differs from the reference human genome at 4.1–5.0 million sites with >99.9% being single nucleotide polymorphisms and short indels (8). This large number of variants can possibly be disease-causing or may contribute to phenotypic variation and drug-response susceptibility. Indeed, a cumulative effect of multiple polymorphisms may lead to complex-trait genetic diseases (8). Here, we hypothesize that variants in the KKS could explain the phenotypic variation observed in HAE.

## Materials and Methods

### Patients

Seventy-eight subjects analyzed by targeted next-generation sequencing (NGS) belonged to 26 C1-INH-HAE families (45 mutation carriers, 15 healthy relatives) and 5 F12-HAE families (14 mutation carriers, 4 healthy relatives) (Table 1). Additional 56 HAE patients were included in the study and had single gene mutations sequenced by Sanger. Patients were previously diagnosed by biochemical C1-INH and C4 measurements and *SERPING1* and/or *F12* genotyping by Sanger sequencing.

**Table 1:** Families analyzed by NGS.

**HAE type**	**Fam ID**	**Nat**	**Mutation leading to HAE**		**Mutation type**	**N^**°**^ symptomatic/asymptomatic**	**Healthy relatives (no mutation)**
			**cDNA change**	**Protein change**			
C1-INH-HAE	1	D	c.-22-2A>G	None	Regulatory region	6/0	1
	2	D	c.23insT	p.(Thr9fs)	Frameshift	1/0	0
	3	B	c.51+1G>T	?	Splicing defect	1/0	0
	4	B	c. 51+2T>C	?	Splicing defect	2/0	0
	5	B	c.97_115del15	p.(Asp33fs)	Frameshift	1/0	0
	6	D	c.143_144delCA	p.(Thr48fs)	Frameshift	2/0	0
	7	D	c.143_144delCA	p.(Thr48fs)	Frameshift	1/0	0
	8	D	c.143_144delCA	p.(Thr48fs)	Frameshift	1/0	0
	9	B	c.550G>C	p.Gly184Arg	Missense	1/0	0
	10	D	c.597C>G	p.Tyr199Ter	Nonsense	1/0	0
	11	D	c.668delA	p.(Gln223fs)	Frameshift	1/0	0
	12	B	c.752T>C	p.Leu251Pro	Missense	1/0	0
	13	D	c.762_763delCA	p.(Asn254fs)	Frameshift	2/0	0
	14	D	c.795G>A	p.Trp265Ter	Nonsense	1/0	0
	15	D	c.838_846del9	p.Ser280_Pro282del	Inframe	1/0	0
	16	D	c.878T>C	p.Ile293Thr	Missense	1/0	0
	17	B	c.889G>A	p.Ala297Thr	Missense	2/0	0
	18	D	c.1029+4delA	?	Splicing defect	1/0	0
	19	B	c.1104delA	p.(Asp369fs)	Frameshift	2/0	0
	20	B	c.1353_1354delGA	p.(Glu451fs)	Frameshift	1/0	1
	21	B	c.1369G>C	p.Ala457Pro	Missense	8/0	13
	22	D	c.1397G>A	p.Arg466His	Missense	1/0	0
	23	D	c.1417G>A	p.Val473Met	Missense	2/0	0
	24	B	c.1431C>A	p.Phe477Leu	Missense	1/0	0
	25	B	c.1480C>T	p.Arg494Ter	Nonsense	2/0	0
	26	D	c.1480C>T	p.Arg494Ter	Nonsense	1/0	0
F12-HAE	27	B	c.983C>A	p.Thr328Lys	Missense	4/2	0
	28	B	c.983C>A	p.Thr328Lys	Missense	3/0	3
	29	B	c.983C>A	p.Thr328Lys	Missense	2/0	0
	30	B	c.983C>A	p.Thr328Lys	Missense	1/0	1
	31	S	c.983C>A	p.Thr328Lys	Missense	2/0	0

*The mutations in SERPING1 and F12 identified as causative of HAE in each family are described. The number of symptomatic and asymptomatic mutation carriers, and healthy relatives (without mutation) analyzed by NGS are shown in the last columns. B, Brazilian; D, Danish; Fam ID, family identification; Nat, nationality; S, Spanish; ?, not known*.

This study was carried out in accordance with the recommendations of ethical standards of the 1964 Declaration of Helsinki with written informed consent from all subjects. The protocol was approved by the Ethics Committee in Research of Universidade Federal de São Paulo (n°56522).

### Genetic Analysis

Since only a few possible genetic variants have been suggested to be associated to the HAE phenotype (9–13), we explored 15 genes related to KKS in individuals from Brazil, Denmark, and Spain, by NGS as previously described (14). Briefly, the targeted genes (*ACE, MME, KNG1, KLKB1, KLK1, F12, ENPEP, BDKRB1, BDKRB2, NOS3, TAC1, CPN1, CPM, SERPING1, PRCP*) were amplified from patients' DNA extracted from blood samples by using Ion AmpliSeq™ Library Kit-2.0V (Life Technologies, Carlsbad, CA, USA). NGS was performed with the Ion 316™ Chip-v2 in Ion PGM™ Sequencer, which determined the amount of sequence data analyzed. To guarantee a high-quality coverage (minimum 20X) we limited the panel to the 15 selected genes, in a total of 366 amplicons. Torrent Suit v3.2.1 and Ion Reporter 4.0 (Life Technologies) were used to data analysis and interpretation of variants. Probably pathogenic variants, low coverage regions and *SERPING1* and *F12* coding regions were validated by Sanger sequencing. Variants annotation was performed using Ingenuity® Variant Analysis (Qiagen) and Metacore™ (Thomson Reuters).

In addition to the 78 subjects evaluated by NGS, in 56 HAE patients the exons 2 and 3 of *BDKRB2* were also sequenced by Sanger method (11 C1-INH-HAE and 45 U-HAE). U-HAE patients were diagnosed according to Cicardi et al.'s guidelines (1). Distribution of variants between groups was calculated by Chi-square test.

All datasets generated and analyzed in this study are included in the manuscript and the Supplementary Files (Tables S1, S2).

### Clinical Characteristics

Patients were grouped and compared according to nationality (Brazilian vs. European), HAE type (C1-INH-HAE vs. F12-HAE), edema localization (face, extremities, abdominal pain, upper respiratory tract, and genitals), mean frequency of attacks (per year) and duration of episodes (days). Patients were categorized in three groups according to the disease onset: (1) < 12-year-old, (2) from 12 to 25-year-old, and (3) >25-year-old. Mean frequency and duration of swelling episodes data were considered at baseline without prophylaxis or acute treatment. The frequency of attacks was divided in low (< 13 attacks per year), medium (from 13 to 24), and high (>24). Patients were also grouped according to the mean duration of swelling episodes in short duration (< 2 days), medium (2–3 days), and long duration (>3 days). Distribution of variants was compared within the selected groups.

## Results

### NGS Analysis

In 26 C1-INH-HAE families, 23 mutations were considered responsible for HAE, and all F12-HAE patients carried p.Thr328Lys (Table 1). A total of 211 different variants were identified in the 15 genes analyzed. Nine alterations occurred in the 5′-UTR (4.3%) and 89 in the 3′-UTR (42.2%). Missense mutations corresponded to 22.7% of the total, followed by synonymous (21.8%), small insertions/deletions leading to frameshift (3.8%), non-sense (1.9%), splice site (1.4%), and intronic alterations (1.9%). A list containing all variants is found in Table S1 (Supplementary Material). *BDKRB2* presented the highest number of variants (*n* = 49), followed by *ACE* (*n* = 26) and *SERPING1* (*n* = 26), and *CPM* (*n* = 24). The distribution of variants per type or per gene was not significantly between C1-INH-HAE and F12-HAE groups (Chi-square test with Yates' correction). Considering only variants leading to change in the structure of the protein (missense, nonsense, indels, and predicted splicing sites), the genes in which more mutations were found were *SERPING1* (*n* = 23) and *ACE* (*n* = 10). No probably damaging variants were identified in *BDKRB1, CPN/M, KNG1, KLK1, ENPEP, PRCP*, or *TAC1* genes. Few studies suggest tissue kallikrein (*KLK1* gene) may be involved in HAE manifestations (15). Tissue kallikrein releases Lys-BK from low molecular weight kininogen (also encoded by *KNG1*), which is converted into BK by aminopeptidases (16) or into Lys-des-Arg9-BK by carboxypeptidases.

### *BDKBR2* Variants

The change p.Arg14Cys in *BDKRB2* was found 15 individuals, and plGly354Glu in 16, without significant difference in distribution among HAE subtypes (*p* = 0.1203 and *p* = 0.1900, respectively, Chi-square test). The alterations p.Val376Met (rs141958164) and p.Val208Ile (rs139203012) were found in one U-HAE patient each.

### Clinical Features and Phenotype-Genotype Association

We found 23 mutations exclusively in Danish individuals and 79 only in Brazilians, resulting in 109/211 mutations in common between European and Brazilian HAE families analyzed.

We compared the symptomatic patients regarding the presence of facial swellings (43/57), swellings of extremities (41/57), abdominal swellings (45/57), upper airways (28/57), and edema of the genitalia (21/57). The first attacks occurred before the age of 12 in 26 patients (24 C1-INH-INH, 2 F12-HAE) (mean = 6.7 years); between 12 and 25 years of age in 21 patients (20 C1-INH-HAE, 5 F12-HAE) (mean = 17), and after 25 years of age in eight (3 C1-INH-HAE, 5 F12-HAE) (mean = 33.8). Most of the patients reported to experience up to 12 swelling episodes/year without prophylaxis (31/47; mean = 4.6). Nine C1-INH-HAE patients reported 13–24 attacks/year (mean = 19.8), and seven reported more than 24 attacks/year (mean = 43.2).

Distribution of variants was compared within the selected groups, but no specific mutation was significantly associated to any of the clinical characteristic selected. However, five missense mutations were classified as probably damaging by all *in silico* predictive software (excluding *SERPING1* variations) (Table 2).

**Table 2:** Probably pathogenic variants found in C1-INH-HAE and F12-HAE patients by NGS.

**Gene**	**Protein change**	**Nat**	**Patient/family**	**Age (years)**	**Onset (years)**	**Frequency (per year)**	**Duration (days)**	**Face**	**Extremities**	**Abdominal**	**Upper airways**	**Genitalia**
*ACE*	p.G354R	B	1/17	49	16	12	3	–	×	–	×	–
		B	2/17	25	NI	< 1	< 1	–	–	×	–	–
		D	1/14	54	10	4	3	×	×	×	×	×
	p.Y244C	D	6/1	73	15	< 1	2–3	×	×	×	–	–
		B	1/24	42	7	2	3	×	×	×	×	×
	p.T916M	D	3/1	71	21	3	2–3	×	×	×	–	–
		D	7/1	36	10	18	3	×	×	×	×	–
*NOS3*	p.D287N	D	5/1	16	3	2	NI	–	–	×	–	–
*KLKB1*	p.C548Y	D	3/1	71	21	3	2–3	×	×	×	–	–
		D	6/1	73	15	< 1	2–3	×	×	×	–	–
		B	4/27	51	Asympt.	Asympt.	Asympt.	Asympt.	Asympt.	Asympt.	Asympt.	Asympt.

*The mutations were analyzed as probably pathogenic when classified as damaging for all the in silico software assessed (SIFT, PolyPhen-2, PROVEAN, and CADD). Asympt, asymptomatic; NI, not identified*.

## Discussion

HAE can be caused by C1-INH deficiency, in which more than 300 different mutations have already been considered responsible for the disease (17). A smaller proportion of HAE patients present specific mutations affecting a proline-rich region of factor XII that leads to loss of glycosylation and enhanced autoactivation mechanism of the zymogen activating the KKS (18). In spite of the large mutational spectrum of *SERPING1* in HAE, the variation in the presentation of symptoms seems not to be related to the type of the disease-causing mutation or even with functional levels of C1-INH (19). Therefore, the search for new genetic biomarkers could not only explain the variation in HAE symptomatology, but also be used in diagnosis, prognosis, and treatment response. Proteins of the KKS are strong candidates to be regulating the severity and frequency of HAE attacks, since they are directly involved in the BK-release pathway.

In this study, a large number of variants was found in a regulatory portion of the 3′-untranslated *BDKRB2* region (20). Also, 40/78 individuals presented the 9 bp deletion in the exon 1 of *BDKRB2*, described to increase B2-receptor expression (21). However, no correlation was found with these variants and HAE clinical characteristics suggesting that the regulation of B2-receptor expression through these regions is not strong enough to modulate HAE attacks itself, in agreement with previous data (22).

Variants classified as probably pathogenic in *ACE* (p.Gly354Arg, p.Tyr244Cys, p.Thr916Met) were found in Brazilian and Danish symptomatic patients, and p.Arg324Trp was found only in one non-affected relative (family 21). Since ACE degrades BK, deleterious alterations could increase BK half-life, favoring longer edema episodes. It has been demonstrated that ACE activity inversely correlates with severity scores in F12-HAE (23); in contrast, C1-INH-HAE severity is not depending on ACE, but highly depends on aminopeptidase P activity (24). However, the C1-INH-HAE patients carrying these mutations had distinguished clinical features from each other (Table 2).

The mutation p.Cys548Tyr in *KLKB1*, found in two patients from family 1 and one asymptomatic mutation carrier from family 27 (F12-HAE), has been associated with KK deficiency in a compound heterozygosity with the change p.Trp402Ter (25). KK deficiency is a rare autosomal recessive condition which causes an augmented activated partial thromboplastin time, but no coagulation anomalies. Therefore, coexistence of KK deficiency could attenuate or even abolish the symptoms of HAE, since it is the key enzyme responsible for BK release. The only missense mutation found together with p.Cys548Tyr was the high frequent polymorphism p.Ser143Asn (66/78). This polymorphism was shown to cause KK deficiency with the change p.Gly125Arg, both in homozygosity (26) and lying in the apple domain 2 of the heavy chain of KK, through which kallikrein interacts with HMWK domain 6 (27). However, p.Ser143Asn was found in heterozygosity in the three patients carrying the p.Cys548Tyr, discarding the hypothesis that this association could be responsible by itself for the lack of symptoms observed in the F12-HAE carrier. Furthermore, the Cys548 lies in the catalytic domain of KK, far from the apple domain 2.

Pathogenicity level of most of the new variants cannot be confirmed only by *in silico* analysis (28). For example, the variant p.Thr328Lys in *F12* is responsible for most of F12-HAE cases (3, 18), but is classified as benign by many pathogenicity predictors. Therefore, the discovery of new and rare variants possibly involved in HAE pathogenesis requires not only experimental evidences to assure the pathogenicity degree of each variant but also the analysis of the overlap effect of different variations and genes. Thus, despite the large number of variants identified in KKS and related genes in this work, the small number of subjects included and the lack of biochemical and functional analysis on the variants are some limitations.

The studies of modulating factors in HAE must combine the discovery of new mutations, genotype-phenotype association including a large number of patients, and consider epigenetic and environmental factors. To our knowledge, this is the largest study exploring different genes in many HAE families. We expect that the disclosure of the variants here presented in HAE patients and the new recent findings in U-HAE will encourage further studies correlating the KKS and the regulation of vasculature, as well as larger international multicentric studies.

## Author Contributions

CV coordinated the analysis, and drafted the manuscript. AA and AB designed data collection. CV, PN, and AM performed all the experiments. CV, RM, and RF-S performed the bioinformatics analysis. AA, RG, JD, MG, AG, EM, and AB performed the data collection instruments, and coordinated data collection. AB conceptualized the study and supervised data collection. JP conceptualized and designed the study, coordinated and supervised all the experiments and analysis and revised the manuscript. All the authors drafted, reviewed and approved the manuscript.

### Conflict of Interest Statement

The authors declare that the research was conducted in the absence of any commercial or financial relationships that could be construed as a potential conflict of interest.

## Supplementary Material

Table S1 Genetic variants found by NGS in HAE families. All the 211 variants found in the 15 genes analyzed in the 78 subjects are listed in the table. Pathogenicity prediction from SIFT (D, damaging; T, tolerated), PolyPhen-2 (B, benign; P, possibly damaging; D, damaging), PROVEAN (D, damaging; N, non-damaging) and CADD (threshold > 20) indicate the *in silico* analysis results. †Found only in Danish individuals.

Table S2 Full dataset of genetic variants and clinical data. The sheet entitled Genetic Variants contains all the variants found by NGS before validation and patients filtering (raw data). Variants with the annotation ARTIFACT in the column Functional Class correspond to the variations not confirmed by Sanger sequencing validation and discarded in the final evaluation. The sheet Patients Raw contains the chip and barcode identification, and clinical data from all the patients initially sequenced, including U-HAE patients posteriorly excluded from NGS analysis.

## Abbreviations

ACE: angiotensin-converting enzyme
C1-INH-HAE: hereditary angioedema with C1 inhibitor deficiency
F12-HAE: hereditary angioedema with mutation in *F12* gene
HMWK: high molecular weight kininogen
KK: Plasma Kallikrein
KKS: Kallikrein-Kinin System
U-HAE: hereditary angioedema of unknown cause.
